# Understanding Breast Cancer: Awareness, Risk Factors, and Symptoms Among Female Health Science Students in Hungary

**DOI:** 10.3390/healthcare13131512

**Published:** 2025-06-25

**Authors:** Sára Garai, Johanna Törzsökné Márton, Melinda Csima, Dávid Sipos

**Affiliations:** 1 Faculty of Health Sciences, Doctoral School of Health Sciences, University of Pécs, 7621 Pécs, Hungary; sgarai.bh@gmail.com (S.G.); mjohanna93@gmail.com (J.T.M.); david.sipos@etk.pte.hu (D.S.); 2 Institute of Education, Hungarian University of Agriculture and Life Sciences, 7400 Kaposvár, Hungary; 3 Department of Medical Imaging, Faculty of Health Sciences, University of Pécs, 7621 Pécs, Hungary

**Keywords:** breast cancer, health science students, risk factors, symptoms, health behavior

## Abstract

**Background**: Breast cancer is the most common malignant neoplasm among women worldwide, and its early detection is crucial for improving survival rates. The aim of our research was to assess the knowledge of health science students regarding breast cancer, with a particular focus on risk factors and symptoms, and to examine their associations with demographic and lifestyle characteristics. **Methods**: A cross-sectional online survey was conducted among 251 female health science students at the University of Pécs. For statistical analysis, we used the Chi-square test, the Mann–Whitney U test, the Kruskal–Wallis test, and Spearman correlation. **Results**: Students were most familiar with the symptoms of breast cancer (59.0%), while knowledge of non-modifiable (44.1%) and lifestyle-related (49.8%) risk factors was found to be lower. Third-year students (H = 15.892; *p* < 0.001), those with better financial status (H = 11.091; *p* = 0.011), physically active individuals (U = 6535.0; *p* = 0.020), and those who regularly performed breast self-examinations (U = 5356.0; *p* = 0.027) achieved significantly higher scores. Knowledge levels also varied by field of study (H = 18.203; *p* = 0.033); students in dietetics and paramedicine stood out with higher results. The majority of students (57.8%) had a moderate level of knowledge, while only 21.9% reached a high level. Surprisingly, the frequency of breast self-examination showed a weak but significant negative correlation with overall knowledge (ρ = −0.155; *p* < 0.05). **Conclusions**: Students’ knowledge requires improvement, particularly regarding risk factors. Targeted education and encouragement of breast self-examination could enhance students’ preparedness, thereby contributing to more effective prevention and early detection.

## 1. Introduction

Breast cancer is a malignant tumor originating from breast tissue characterized by the uncontrolled proliferation of cells, which can infiltrate surrounding tissues and form distant metastases, leading to severe and potentially life-threatening complications. Its high morbidity and mortality rates are primarily attributable to the fact that it is typically asymptomatic in its early stages, resulting in delayed diagnosis and frequent detection at an advanced stage of the disease. Due to this characteristic, breast cancer is often referred to as a “silent killer” [1,2,3].

In 2022, breast cancer was the second most common type of cancer globally among women, with 2.3 million new cases, accounting for 11.6% of all cancer diagnoses, and it was the fourth leading cause of cancer-related death, with 666,000 deaths. Globally, breast cancer was the most frequently diagnosed cancer type among women and the leading cause of cancer death in 112 countries [4]. The goal of the World Health Organization’s Global Breast Cancer Initiative (GBCI) is to reduce global breast cancer mortality by 2.5% annually, thereby saving 2.5 million lives. If the global annual mortality rate were to decrease by 2.5%, this could result in the prevention of 25% of breast cancer deaths by 2030 and 40% by 2040 among women under the age of 70 [5,6].

In Hungary, breast cancer has also become one of the most common malignant diseases among women. Between 2011 and 2020, the incidence of breast cancer among women over the age of 50 showed a continuous increase. The age-standardized incidence rate (ASIR) per 100,000 population was 152.7 in 2011, rising to 162.6 by 2019. The annual percentage change (APC) in this age group was significant, showing a yearly increase of 0.7% [7]. According to the most recent available statistics, in 2022, a total of 7694 new breast cancer cases were registered in Hungary, and 2237 women died as a result of the disease—representing a mortality rate of 29.1% [8]. In Hungary, breast cancer is currently the third most common cause of cancer-related death, which is similar to trends observed in more developed countries. In order to reduce the societal and healthcare burden caused by cancer, significant progress has been made in Hungary in the fields of diagnostics and oncological therapy. Organized cancer screening programs have been available in the country since 2001 [9].

Breast cancer is a disease with a multifactorial etiological background in which numerous endogenous and exogenous factors play a role [10,11,12]. Risk factors can be classified into two main categories: modifiable and non-modifiable factors. Proper control of the former, along with lifestyle interventions, can significantly contribute to the prevention of the disease [13]. Genetic predisposition plays a significant role in the development of breast cancer. Carriers of BRCA1 and BRCA2 gene mutations face a substantially increased risk of both breast and ovarian cancer, with a lifetime risk of up to 85% [14,15]. A positive family history further increases the likelihood of developing the disease [16]. Hormonal factors include late menopause (after the age of 55) and nulliparity, both of which are associated with an elevated risk [17,18]. Hormone replacement therapy and the use of oral contraceptives also increase risk, although this risk decreases after discontinuation of the treatment [19,20]. Postmenopausal obesity is another significant factor, as adipose tissue contributes to estrogen production, which in turn promotes breast tissue proliferation [21,22]. Among environmental factors, radiation therapy administered at a young age increases the risk of breast cancer [23], while regular physical activity has been shown to reduce its incidence [24]. Lifestyle factors, such as alcohol consumption and smoking, also elevate risk [25]. Nutrition plays a noteworthy role as well; diets high in saturated fats and processed foods increase risk, whereas a diet rich in antioxidants and fiber may have a protective effect [26,27].

University students are at a critical stage in life where long-term health behaviors are shaped and health-related autonomy increases. Despite the advantages of the academic environment, international studies indicate that breast-cancer-related knowledge in this population remains limited [28,29,30,31,32]. While numerous studies have examined breast cancer knowledge, risk factor awareness, and symptom recognition worldwide, no comprehensive research has yet been conducted in Hungary among female university students. Addressing this gap is essential, as the university setting provides a valuable opportunity to foster health awareness and establish preventive behaviors early on. Moreover, to our knowledge, no previous study has specifically focused on this age group to assess their knowledge of breast cancer, particularly regarding symptom recognition, lifestyle-related risks, and non-modifiable risk factors. The focus on health science students is especially relevant, as they represent the future healthcare workforce and are expected to serve as role models in promoting public health. Even during their studies, they have the potential to initiate health promotion activities and disseminate preventive messages. Therefore, it is particularly important to assess their current knowledge and attitudes regarding breast cancer prevention, as these factors may indirectly influence the effectiveness of future health education and disease prevention strategies.

While international research highlights limited knowledge among university students, Hungarian data also suggest that even professionals responsible for health education exhibit significant differences in health behaviors and attitudes toward screening participation. A previous study conducted in Hungary [33] examined health status, health behaviors, and willingness to participate in screening programs among professional groups whose key responsibilities include health education and health promotion. Regarding participation in mammography screening, the study found that nurses were significantly less willing to participate compared to early childhood educators. While nurses may influence the health-related decisions of the general population, early childhood educators shape the health behaviors of future generations. The results clearly highlight the critical importance of shaping the attitudes of current and future healthcare professionals.

In light of all of this, it is especially important that future healthcare professionals possess adequate knowledge of breast cancer risk factors, symptoms, and prevention strategies. The aim of the present study was to assess the breast-cancer-related knowledge of health science students at the University of Pécs, with a particular emphasis on symptoms, as well as lifestyle-related and non-modifiable risk factors. The purpose of the research was to map the students’ level of knowledge so that the results may inform the development of targeted educational programs to support their future clinical practice.

## 2. Materials and Methods

A cross-sectional online survey was conducted using a non-random sampling method. The study took place at the Faculty of Health Sciences, University of Pécs, between September and November 2024. The research received ethical approval from the Scientific and Research Ethics Committee of the Health Science Council (BM/23045-3/2024). The study included only female students who were at least 18 years old and enrolled in undergraduate programs at the Faculty of Health Sciences, University of Pécs, and who voluntarily consented to participate. Male students, as well as those enrolled in master’s or doctoral (PhD) programs, were not included in the study. Additional exclusion criteria included a personal history of breast cancer or currently undergoing treatment for breast cancer.

At the close of the survey, a total of 252 responses were received; however, 1 respondent was excluded for not meeting the inclusion criteria. As a result, 251 participants were included in the final analysis. The sampling method used was convenience sampling. The questionnaire was developed by adapting several previously published instruments [34,35,36,37,38,39,40]. Completion of the questionnaire was voluntary and anonymous, and participants received detailed information about the purpose and procedures of the study prior to participation. The response rate among participants was 100%. The Cronbach’s alpha value was 0.754 for the items assessing non-modifiable risk factors, 0.777 for lifestyle-related risk factors, and 0.736 for symptom-related items, all indicating acceptable internal consistency.

The questionnaire was created using Google Forms and distributed to students via the Teams application. Completion took approximately 15–20 min. On the first page of the questionnaire, it was stated that the research served scientific purposes and that all data would be treated confidentially. Only students who selected the “I have read and agree” option were allowed to participate. To prevent multiple submissions, the Google Forms “Limit to 1 response” setting was applied.

The questionnaire consisted of 44 questions divided into five sections: socio-demographic data (15 questions), including age, university year and field of study, subjective assessment of financial status, level of trust in the healthcare system and physicians, level of physical activity, alcohol consumption habits, family medical history, awareness and attitudes regarding cancer, frequency of breast self-examination (BSE), and the date of the last self-examination. In addition, the questionnaire included items assessing knowledge of breast cancer symptoms and signs (8 questions) and knowledge of risk factors (20 questions), and it asked participants to indicate their main sources of information related to breast cancer (1 question).

Statistical analyses were performed using IBM SPSS (Statistical Package for Social Sciences) version 27.0.1. Descriptive statistics included the calculation of frequency distributions, percentages, means, and standard deviations. Variables were reported as mean ± standard deviation (SD), median and interquartile range (IQR), count, and percentage. To examine associations between nominal variables, the Chi-square test was applied. The normality of continuous variables was assessed using the Kolmogorov–Smirnov test. Because most of the data did not follow a normal distribution, the Mann–Whitney U test was used for comparing two groups, and the Kruskal–Wallis test was applied for comparing more than two groups. Relationships between variables were evaluated using Spearman’s rank correlation coefficient. A *p*-value of <0.05 was considered statistically significant. In the assessment of knowledge, correct answers were awarded 1 point, while incorrect answers received 0 points. The average percentage knowledge score was calculated using the following formula: (total score achieved/maximum possible score) × 100. Knowledge levels were categorized into three groups based on the percentage of correct answers: scores ranging from 0 to 33.3% were classified as indicating a low level of knowledge, scores between 33.4% and 66.6% were considered moderate, and scores from 66.7% to 100% represented a high level of knowledge. This classification was adapted from the methodology used in two previous studies [41,42].

## 3. Results

The demographic characteristics of the students participating in the study are presented in Table 1. A total of 251 students participated, with a median age of 21 years (IQR: 20–23). The majority of participants (84.1%) were between the ages of 18 and 29. Of the students, 28.7% were in their first year, 28.3% were in their second year, and 43.0% were in their third year. The most common fields of study were physiotherapy (33.5%), nursing (15.5%), and radiography (11.6%). Most students rated their financial status as “good” (52.2%) or “excellent” (14.7%). Trust in the healthcare system and physicians was reported as moderate (62.1%) or low (27.1%). A vast majority (96.8%) of students considered information about cancer to be important. Physical activity was reported by 47.0% of respondents, and 84.1% consumed alcohol. A family history of breast cancer was reported by 45.0% of participants. BSE was performed by 70.9%, and 50.2% had performed it within the past month. Additional characteristics of the participants are presented in Table 1.

Regarding knowledge of non-modifiable risk factors, a substantial portion of respondents were aware of the role of inherited gene mutations (82.1%) and family history (88.0%). However, fewer recognized race/ethnicity (25.9%) and greater height (13.5%) as potential risk factors. In terms of lifestyle-related risk factors, the use of oral contraceptives (68.1%), being overweight or obese (64.9%), and alcohol consumption (61.0%) were the most widely recognized. However, fewer participants were aware that nulliparity (19.1%) and not breastfeeding (28.3%) are also associated with increased risk. Overall, students demonstrated greater knowledge of breast cancer symptoms compared to risk factors. A palpable lump in the breast was the most widely recognized symptom, identified by 97.2% of respondents, indicating strong awareness of this classic warning sign. This widespread recognition is likely influenced by school-based education and public health messaging through media. However, awareness of less obvious symptoms was considerably lower; only 41.0% correctly identified nipple retraction, and 43.8% recognized skin changes as potential signs of breast cancer. A detailed distribution of responses is presented in Table 2.

The average knowledge scores across the three assessed areas varied and are illustrated in Table S1. For non-modifiable risk factors, the mean score was 5.29 (SD = 2.79) out of a maximum of 12 points; for lifestyle-related risk factors, the mean score was 3.98 (SD = 1.81) out of 8; and for symptoms, the average score was 4.72 (SD = 2.16) out of 8 points. The proportion of correct answers was 44.1% for non-modifiable risk factors, 49.8% for lifestyle-related risk factors, and the highest for symptom knowledge at 59.0%. In terms of lifestyle-related risk factor knowledge, students aged 30–41 scored significantly higher than both younger and older groups (H = 6.946; *p* = 0.031). Based on university year, third-year students had the highest level of knowledge, while first-year students had the lowest scores (H = 15.892; *p* < 0.001).

Our findings showed that several demographic and lifestyle factors were significantly associated with breast-cancer-related knowledge. Among fields of study, knowledge of lifestyle-related risk factors differed significantly (H = 18.203; *p* = 0.033); students majoring in dietetics and paramedicine achieved the highest scores, while those studying nursing and medical laboratory analysis had the lowest. Knowledge of non-modifiable risk factors was also significantly higher among students who reported better financial status (H = 11.091; *p* = 0.011). The level of trust in the healthcare system and physicians also influenced knowledge; students with a moderate level of trust scored higher in this area (H = 7.163; *p* = 0.028). Participants who strongly agreed with the statement that they would want to know if they had cancer also performed better in terms of knowledge of non-modifiable risk factors (H = 10.876; *p* = 0.004).

Regarding lifestyle-related risk factors, physically active students scored significantly higher than their inactive peers (U = 6535.0; *p* = 0.020), while those who consumed alcohol had lower scores in this domain (U = 5040.0; *p* = 0.015). Family history also had an impact; participants with a positive family history of breast cancer demonstrated better knowledge of non-modifiable risk factors (U = 6526.5; *p* = 0.025). Additionally, students who regularly performed BSE had significantly higher knowledge levels both in non-modifiable risk factors (U = 5356.0; *p* = 0.027) and recognizing breast cancer symptoms (U = 5341.5; *p* = 0.025). Those who had performed a self-examination within the past month also achieved higher scores in knowledge of non-modifiable risk factors (U = 6655.5; *p* = 0.032).

Table 3 presents the correlations between variables as measured by Spearman’s rank correlation coefficient. Knowledge of non-modifiable risk factors showed a significant positive correlation with knowledge of lifestyle-related risk factors (ρ = 0.451, *p* < 0.01), symptom recognition (ρ = 0.488, *p* < 0.01), and overall knowledge (ρ = 0.828, *p* < 0.01). A positive correlation was also found between knowledge of lifestyle-related risk factors and symptom recognition (ρ = 0.330, *p* < 0.01), while a strong correlation was observed between lifestyle-related risk factors and overall knowledge (ρ = 0.714, *p* < 0.01). The relationship between symptom recognition and overall knowledge was also strong and positive (ρ = 0.780, *p* < 0.01). However, the practice of BSE showed a negative correlation with several knowledge domains, including symptom recognition (ρ = –0.141, *p* < 0.05) and overall knowledge (ρ = –0.155, *p* < 0.05).

Multiple linear regression analyses were conducted to assess the effects of age, years of university study, perceived financial status, and physical activity on breast cancer knowledge across different domains. The regression model predicting overall knowledge was statistically significant (R^2^ = 0.055, *p* = 0.008). Within this model, years of university studies (B = 1.186, *p* = 0.004) and perceived financial status (B = 1.067, *p* = 0.025) emerged as significant positive predictors. Similarly, the model for knowledge of non-modifiable risk factors was also significant (R^2^ = 0.054, *p* = 0.009), with both years of university studies (B = 0.438, *p* = 0.037) and perceived financial status (B = 0.763, *p* = 0.002) showing significant associations. The model related to lifestyle-related risk factor knowledge demonstrated the highest explanatory power (R^2^ = 0.088, *p* < 0.001). In this domain, both years of university studies (B = 0.486, *p* < 0.001) and physical activity (B = 0.508, *p* = 0.022) were significant predictors. In contrast, the model predicting symptom knowledge was not statistically significant (R^2^ = 0.026, *p* = 0.164), although age group showed a marginally significant effect (B = 0.534, *p* = 0.032). Detailed regression coefficients are presented in Table 4.

Table 5 presents the distribution of participants’ knowledge levels across different areas related to breast cancer. Knowledge of non-modifiable risk factors was the least developed; nearly half of the respondents (49.0%) fell into the low knowledge category. The proportion of those with moderate knowledge was 36.3%, while only 14.7% had a high level of knowledge. The median score in this domain was just 2.0, reflecting a low level of understanding. Knowledge related to lifestyle risk factors was somewhat higher, as 22.3% of participants had low, 58.2% moderate, and 19.5% high knowledge, with a median score of 4.0. The best results were observed in the recognition of breast cancer symptoms, as 17.5% had low, 47.8% moderate, and 34.7% high knowledge, with a median score also of 4.0. Based on overall knowledge scores, the majority of participants fell into the lower categories, as 20.3% had low, 57.8% moderate, and only 21.9% high knowledge. The average overall knowledge score was 13.99 ± 5.40, with a median of 13.0, indicating that most participants belonged to the moderate knowledge group.

Table 6 illustrates the relationship between sources of information and knowledge levels. Most participants reported obtaining information from personal connections (61.4%) and online sources (52.6%). Education (42.6%) and healthcare professionals (39.4%) also played significant roles, while the fewest participants relied on media and printed sources (24.7%). These results suggest that personal and digital sources dominate. Use of media and printed sources was associated with significantly higher knowledge in both non-modifiable risk factors (5.5 vs. 4.0, *p* = 0.009, χ^2^ = 9.391) and lifestyle-related risk factors (4.0 vs. 3.0, *p* = 0.014, χ^2^ = 8.526). Participants who used online sources also scored higher on non-modifiable risk factors (5.0 vs. 4.0), although the difference was not statistically significant (*p* = 0.090, χ^2^ = 4.824). Information obtained through personal connections had a positive impact on overall knowledge (13.0 vs. 13.0, *p* = 0.031, χ^2^ = 6.972). Information from healthcare professionals and online sources did not show a significant effect. Knowledge gained through formal education showed a significant difference in the area of non-modifiable risk factors (5.0 vs. 4.0, *p* = 0.004, χ^2^ = 11.050) but not in other domains. Despite the dominance of informal sources, such as personal connections and online media, structured educational content and traditional printed materials appear to play a more significant role in improving knowledge of breast cancer risk factors.

## 4. Discussion

The aim of the present study was to assess breast-cancer-related knowledge among female students at the Faculty of Health Sciences, University of Pécs, with a particular focus on symptoms and both modifiable and non-modifiable risk factors. Based on the findings, the students’ overall knowledge level fell into the lower category; however, significant differences were observed depending on year of study, field of specialization, and BSE practices.

One of the key findings of our research was that third-year students had significantly higher levels of breast cancer knowledge compared to students in earlier years. This is consistent with previous studies [28,43], which also support the notion that academic progression plays an important role in enhancing breast-cancer-related knowledge. A study from East Africa similarly concluded that participants with a Bachelor’s degree demonstrated significantly greater knowledge than those with only a diploma-level education [29]. These differences can partly be attributed to the students’ health sciences education, where clinical and preventive knowledge increases with academic advancement. A proportionally higher number of third-year students were enrolled in diagnostic imaging and dietetics programs, where breast disease education is emphasized more, likely contributing to their higher knowledge levels. As breast cancer education varies by specialization, it is recommended that curricula be revised to ensure all students—regardless of their field—receive basic knowledge and practical training, with a particular focus on BSE. These differences across disciplines likely reflect variations in curricular priorities and the extent to which breast health education is integrated into the training of different professional groups. For example, programs like dietetics and diagnostic imaging may include more targeted content related to cancer prevention, screening techniques, and anatomical knowledge. In contrast, other fields may offer less comprehensive or more general health education, resulting in lower levels of topic-specific knowledge. Understanding these structural differences is essential for identifying gaps and ensuring that all health science students, regardless of specialization, are equally prepared to engage in breast cancer prevention and patient education.

Additional studies have confirmed the link between education and breast cancer knowledge. For instance, an Indian study [30] found that women with more than ten years of formal education were nearly four times more likely to possess adequate breast cancer knowledge compared to those with less than ten years of education. This suggests that disparities between institutions and training programs may be due to differences in curricular structure. Therefore, it is essential that all female university students, regardless of specialization, have access to fundamental breast cancer knowledge, thus supporting the promotion of screening and the development of preventive behaviors.

Although the association between year of study and knowledge level was anticipated, it is important to critically examine the curricular and pedagogical factors that may underlie this pattern. In many health science programs, breast-cancer-related content is not evenly distributed across academic years or disciplines. Earlier-year students may receive less exposure to clinical practice or preventive education, which could contribute to lower knowledge levels. Furthermore, pedagogical approaches like problem-based learning, clinical simulations, and integrated case discussions, which are typically introduced in more advanced stages of education, may play a key role in deepening understanding and promoting long-term knowledge retention. These findings suggest that curriculum designers should carefully consider both the timing and the instructional methods used in breast health education, ensuring that essential content is introduced at an earlier stage and reinforced consistently throughout the educational process.

When comparing our findings to those of other studies, significant differences emerge in the level of breast cancer knowledge and the practice of self-examination. Among Hungarian students, knowledge of breast cancer symptoms reached 59%, which is lower than the highest values recorded in Indonesian and Pakistani student populations (87.6%) but exceeds the lowest level observed (46.2%) [31].

In our sample, Hungarian students demonstrated a moderate level of knowledge regarding breast cancer symptoms, with nearly half falling into the mid-range and over one-third showing high awareness. This overall result reflects a satisfactory level of symptom recognition, surpassing the outcomes reported at King Edward Medical University but falling short of the notably high performance observed among students at KSAU-HS. While symptom knowledge was relatively strong, understanding of non-modifiable risk factors was considerably weaker, with only a small proportion of students reaching a high level. Compared to international findings, such as the higher scores reported among Iranian students, Hungarian students’ awareness in this domain appears limited. In contrast, knowledge of lifestyle-related risk factors proved more favorable and even exceeded the scores reported at both King Edward University and KSAU-HS, suggesting that students are more attuned to modifiable health behaviors. Overall, 20.3% of Hungarian students had low, 57.8% moderate, and 21.9% high levels of knowledge. This is better than the 33.3% high knowledge level reported at King Abdulaziz University but lower than the 64.1% high knowledge level among the Iranian sample [32,44,45,46].

Recognition of family history as a risk factor was consistently high across all countries included in the comparison, with the strongest awareness observed among Saudi Arabian students, followed closely by Hungarian and Pakistani students. Awareness of older age as a risk factor was again most prominent in Saudi Arabia, moderately acknowledged in Hungary, and surprisingly low among students in Pakistan. Alcohol consumption as a risk factor was well-recognized in both Saudi Arabia and Hungary but received considerably less attention in the Pakistani sample. Overweight and obesity were most commonly identified as risk factors in Hungary and, to a slightly lesser extent, at Saudi institutions, while Pakistani students showed lower awareness in this area. Similarly, the risks associated with physical inactivity were more frequently acknowledged in Hungary and Saudi Arabia than in Pakistan. The use of oral contraceptives was widely recognized as a lifestyle-related risk factor in Hungary and at King Abdulaziz University but received less emphasis in the other Saudi institution. In the case of early menarche, students at King Abdulaziz University demonstrated the highest level of awareness, followed by those at KSAU-HS, Hungarian students, and, lastly, Pakistani students [32,44,45].

The regression analysis of differentiating factors related to the breast cancer knowledge domains—consistent with the findings of previous research [28,29,30,32,43,44,45,46]—identified years of university study and perceived financial status as key predictors of breast cancer knowledge, particularly in relation to overall understanding and non-modifiable risk factors. Additionally, higher levels of physical activity were linked to greater awareness of lifestyle-related risk factors. These findings highlight that both socioeconomic and behavioral factors contribute to variations in specific domains of breast cancer knowledge.

The occurrence of breast cancer symptoms in Pakistan and Hungary shows a similar pattern, with minor differences. In both countries, the most commonly recognized symptom was a palpable lump in the breast (Pakistan 84.6% vs. Hungary 97.2%). Axillary lumps (66.2% vs. 75.7%) and breast or underarm pain (71.1% vs. 62.5%) were also frequently noted. However, nipple retraction (63.9% vs. 41.0%) and changes in nipple position (70.7% vs. –) were more commonly recognized in Pakistan. Skin-related symptoms, such as dimpling or wrinkling (79.3% vs. 37.5%) and redness/dryness/peeling (39.1% vs. 43.8%), also showed some variation [32]. This comparison indicates that although the overall pattern of symptom recognition is similar between the two countries, Pakistani students appear to be more aware of less commonly recognized or visual breast cancer symptoms than their Hungarian peers.

Although a strong positive correlation was found between symptom recognition and overall knowledge, BSE practice showed a weak but significant negative correlation with these knowledge variables. This indicates that a higher level of knowledge alone does not guarantee regular practice of BSE. Psychological and social factors, such as fear, anxiety, or lack of confidence, may influence the frequency of self-examinations and should be taken into account in health education programs.

Regarding sources of information, most participants cited personal relationships as their primary source, followed by the internet and education. This is consistent with findings from other studies, which highlight the crucial role of mass media, particularly online sources, in disseminating health information. Prior research has emphasized the importance of social media, television, and radio in information delivery [47,48], and our findings support this, as well. Nevertheless, personal networks and educational institutions also play a significant role in acquiring breast-cancer-related knowledge.

Interestingly, the results of our study differ from other research involving health science students, where university studies were cited as the primary source of information, followed by the internet and social media [49]. Studies from Nigeria and Hungary found that the majority of medical students acquired their knowledge of breast cancer through curricular education [50,51]. In contrast, our findings suggest that even students enrolled in health science programs primarily obtain information from personal sources (61.4%) and online platforms (52.6%), while education (42.6%) and healthcare professionals (39.4%) play a secondary role. This indicates a preference for easily accessible, informal sources over formal educational materials.

Our study also suggests that students who regularly perform BSE have higher knowledge levels, particularly when the self-examination is performed at the recommended time. This aligns with earlier studies [52,53] that have confirmed a positive relationship between BSE-related knowledge and attitudes. Our findings further support the notion that proper education and awareness campaigns can encourage the regular practice of BSE, which plays a crucial role in early detection of breast cancer. Building on these findings, it is important to consider not only the content of educational interventions but also the methods through which information is delivered. Additionally, visual learning tools, such as educational videos, animated infographics, and mobile-based tutorials, should be embedded in curricula to reinforce engagement, improve knowledge retention, and enhance procedural confidence. Prior studies have confirmed the value of such materials in health education, particularly in increasing BSE-related understanding and self-efficacy [54,55,56,57,58].

An important observation from this study is the lack of a consistent association between knowledge level and the frequency of BSE. While many students demonstrated adequate knowledge, this did not consistently translate into regular practice. This knowledge–behavior gap may be explained by a range of psychosocial barriers, such as fear of illness, low self-efficacy, and distrust in screening methods, that inhibit action despite awareness. This phenomenon is consistent with both the Health Action Process Approach (HAPA) and the Health Belief Model (HBM). According to the HAPA, behavior change requires more than knowledge; factors like risk perception, outcome expectancies, action planning, and coping strategies also play a role [59]. Similarly, the HBM highlights the importance of perceived susceptibility, perceived severity, perceived benefits, and perceived barriers in shaping preventive health behaviors [60,61]. Together, these models underscore that knowledge is a necessary but insufficient condition for behavior change, which is instead shaped by a complex interplay of cognitive, emotional, and contextual factors.

Assessing the knowledge of future healthcare professionals is essential, as it impacts the quality of patient care, the effectiveness of prevention programs, and the development of the healthcare system. Well-trained professionals not only enhance patient care but also contribute to prevention, early detection, and effective patient education, thereby supporting the sustainability and efficiency of the healthcare system [54]. The findings of this study highlight the need to revise curricula and more strongly integrate breast cancer prevention knowledge into health science education. In addition, the educational potential of online platforms and social media should be utilized in a more structured and intentional way to ensure that students receive accurate and reliable information from trustworthy sources. Future research should consider longitudinal follow-up studies to better understand which factors most effectively support long-term knowledge retention and practical application regarding breast cancer.

## 5. Limitations

This study has several limitations that may affect the generalizability and reliability of the results. First, the research was limited to students enrolled in health science programs at a single university, and thus the findings may not be representative of all health science faculties in Hungary. Secondly, the study included only female students, who were predominantly young and currently enrolled in higher education, which limits the applicability of the results to women of different ages or from varied social backgrounds. Another limitation is that the data were collected through a self-reported questionnaire, without the use of objective measurement tools. This may introduce bias, as respondents may be inclined to provide socially desirable answers. Moreover, all symptoms and risk factors listed in the questionnaire were in fact associated with breast cancer, which may have caused uncertainty among participants, potentially leading to underreporting of correct answers. It is also possible that unmeasured confounding variables, such as prior exposure to breast health education, family history of breast cancer, or personal health experiences, may have influenced the responses. Additionally, subjective interpretations of questionnaire items could vary between participants, further limiting the accuracy of the self-reported data. Finally, due to the cross-sectional design of the study, causal relationships cannot be established; therefore, the results reflect correlations rather than cause-and-effect conclusions.

## 6. Conclusions

Our research highlighted that a significant proportion of students enrolled in health science programs lack adequate knowledge about critical risk factors, such as early menarche, nulliparity, and the absence of breastfeeding. Another major gap identified was the insufficient recognition of less well-known but diagnostically important symptoms, such as nipple retraction and skin changes in the breast, which are crucial for early detection.

Analysis of the relationship between knowledge and self-examination behavior revealed that possessing adequate knowledge does not necessarily translate into more conscious health behaviors. This suggests that cognitive awareness alone does not automatically lead to preventive behaviors. It points to the need for educational programs that address not only informational content but also psychological and motivational determinants of health behavior, such as perceived self-efficacy, social norms, and emotional barriers.

Building on our findings, we recommend that educational programs devote increased attention to underrepresented risk factors, such as early menarche, nulliparity, and lack of breastfeeding, by delivering focused, evidence-based content. To support the development of effective BSE habits, training should go beyond theoretical instruction and incorporate experiential learning approaches, including guided BSE demonstrations, hands-on practice, and peer-led workshops. We also propose the implementation of longitudinal follow-up studies that evaluate not only the retention of breast-cancer-related knowledge but also the frequency, quality, and confidence in BSE over time. These studies should compare different instructional formats (e.g., video-based learning vs. instructor-led sessions) to identify the most effective methods for sustaining preventive health behaviors.

## Figures and Tables

**Table 1:** Demographic characteristics and health-related attitudes of students (n = 251).

Variables	n (%)
Age (median, IQR)	21 (20–23)
**Age group**	
18–29	211 (84.1)
30–41	23 (9.2)
42–53	17 (6.8)
**Year of university studies**	
First year	72 (28.7)
Second year	71 (28.3)
Third year	108 (43.0)
**Field of specialization**	
Nursing	39 (15.5)
Dietetics	25 (10.0)
Physiotherapy	84 (33.5)
Midwifery	28 (11.2)
Paramedicine	9 (3.6)
Public Health Inspector	4 (1.6)
Health Visitor	17 (6.8)
Recreation and Lifestyle	2 (0.8)
Medical Laboratory Diagnostics	14 (5.6)
Radiography	29 (11.6)
**Perceived financial status**	
Poor	6 (2.4)
Just sufficient	77 (30.7)
Good	131 (52.2)
Excellent	37 (14.7)
**Trust in the healthcare system and physicians**	
Low	68 (27.1)
Moderate	156 (62.1)
High	27 (10.8)
**“If I had cancer, I would want to know about it.”**	
Not sure	8 (3.2)
Agree	74 (29.5)
Strongly agree	169 (67.3)
**Physical activity**	
Yes	118 (47.0)
No	133 (53.0)
**Alcohol consumption**	
Yes	211 (84.1)
No	40 (15.9)
**Family medical history**	
Negative	138 (55.0)
Positive	113 (45.0)
**Performs BSE**	
Yes	178 (70.9)
No	73 (29.1)
**Performed BSE in the last month**	
Yes	126 (50.2)
No	125 (49.8)

**Table 2:** Students’ knowledge of non-modifiable and lifestyle-related risk factors and symptoms of breast cancer (N = 251).

Variables	n (%)
Non-Modifiable Risk Factors	
Being born female	150 (59.8)
Aging	112 (44.6)
Inherited genetic mutations	206 (82.1)
Family history of breast cancer	221 (88.0)
Previous personal diagnosis of breast cancer	130 (51.8)
Race and ethnicity	65 (25.9)
Greater height	34 (13.5)
Dense breast tissue	92 (36.7)
Certain benign breast conditions	91 (36.3)
Early onset of menstruation (<12 years)	67 (26.7)
Late menopause (>55 years)	70 (27.9)
High-dose radiation to the chest	90 (35.9)
Lifestyle-Related Risk Factors	
Alcohol consumption	153 (61.0)
Overweight/obesity	163 (64.9)
Physical inactivity	154 (61.4)
Nulliparity	48 (19.1)
Not breastfeeding	71 (28.3)
Use of oral contraceptives	171 (68.1)
Hormone therapy during menopause	118 (47.0)
Breast implants	121 (48.2)
Symptoms	
Palpable lump in the breast	244 (97.2)
Swelling of all or part of the breast	155 (61.8)
Dimpling or puckering of the breast skin	94 (37.5)
Pain in the breast and/or nipple	157 (62.5)
Nipple retraction (inversion)	103 (41.0)
Redness, dryness, flaking, or thickening of the skin	110 (43.8)
Nipple discharge	132 (52.6)
Swollen lymph nodes in the armpit or near the collarbone	190 (75.7)

**Table 3:** Descriptive statistics and Spearman correlations between outcome variables.

Variables	Kurtosis (SE)	Skewness (SE)	Mean (SD)	Range	1	2	3	4	5
Non-modifiable risk factors	−1.083 (0.306)	0.146 (0.154)	5.290 (2.790)	1–12	⸻	0.451 **	0.488 **	0.828 **	−0.139 *
Lifestyle risk factors	−0.407 (0.306)	0.491 (0.154)	3.980 (1.810)	1–8	0.451 **	⸻	0.330 **	0.714 **	−0.098
Symptoms	−1.151 (0.306)	0.927 (0.154)	4.720 (2.160)	1–8	0.488 **	0.330 **	⸻	0.780 **	−0.141 *
Overall knowledge	−0.401 (0.306)	0.566 (0.154)	13.990 (5.400)	3–28	0.828 **	0.714 **	0.780 **	⸻	−0.155 *
BSE practice	0.207 (–)	1.291 (0.029)	1.290 (0.460)	1–2	−0.139 *	−0.098	−0.141 *	−0.155 *	⸻

* Correlation is significant at the 0.05 level (2-tailed). ** Correlation is significant at the 0.01 level (2-tailed).

**Table 4:** Results of multiple linear regression analyses of factors associated with breast cancer knowledge domains.

Knowledge Domain	B	ANOVA *p*-Value	R^2^	F (df)	*p*-Value
**Significant Predictor(s)**					
**Non-modifiable risk factors**					
Years of university studies	0.438	0.037	0.054	F (4.246) = 3.493	0.009
Perceived financial status	0.763	0.002			
**Lifestyle-related risk factors**					
Years of university studies	0.486	<0.001	0.088	F (4.246) = 5.899	<0.001
Physical activity	0.508	0.022			
**Symptom knowledge**					
Age group	0.534	0.032	0.026	F (4.246) = 1.643	0.164
**Overall knowledge**					
Years of university studies	1.186	0.004	0.055	F (4.246) = 3.569	0.008
Perceived financial status	1.067	0.025			

**Table 5:** Participants’ scores in the three breast cancer knowledge subdomains by quartile (N = 251).

Score	Knowledge Subdomain			
Quartile	Non-Modifiable Risk Factors n (%)	Lifestyle Risk Factors n (%)	Symptoms n (%)	Overall Knowledge n (%)
Low (Q1)	123 (49.0)	56 (22.3)	44 (17.5)	51 (20.3)
Moderate (Q2)	91 (36.3)	146 (58.2)	120 (47.8)	145 (57.8)
High (Q3)	37 (14.7)	49 (19.5)	87 (34.7)	55 (21.9)
Mean ± SD	5.29 ± 2.79	3.98 ± 1.81	4.72 ± 2.16	13.99 ± 5.40
Median	2.0	4.0	4.0	13.0
Minimum–maximum	1–12	1–8	1−8	3–28
Range	11	7	7	25

**Table 6:** Association between different sources of information and knowledge levels based on Chi-square test.

Source of Information												Knowledge Scores, Median (Min–Max)		
		n	Non-Modifiable Risk Factors	*p* Value	Pearson Chi-Square	Lifestyle Risk Factors	*p* Value	Pearson Chi-Square	Symptoms	*p* Value	Pearson Chi-Square	Overall Knowledge	*p* Value	Pearson Chi-Square
Media and printed sources	Yes	62	5.5 (1–12)	0.009 *	9.391	4.0 (1–8)	0.014 *	8.526	5.0 (1–8)	0.369	1.996	15.0 (3–27)	0.127	4.134
	No	189	4.0 (1–12)			3.0 (1–8)			4.0 (1–8)			12.0 (3–28)		
Online sources	Yes	132	5.0 (1–12)	0.090	4.824	4.0 (1–8)	0.210	3.117	4.0 (1–8)	0.199	3.232	13.0 (3–27)	0.462	1.546
	No	119	4.0 (1–12)			4.0 (1–8)			5.0 (1–8)			13.0 (5–28)		
Personal contacts	Yes	154	5.0 (1–12)	0.445	1.620	4.0 (1–8)	0.398	1.844	4.0 (1–8)	0.205	3.171	13.0 (3–27)	0.031 *	6.972
	No	97	5.0 (1–12)			4.0 (1–8)			5.0 (1–8)			13.0 (3–28)		
Healthcare professionals	Yes	99	5.0 (1–12)	0.385	1.908	4.0 (1–8)	0.669	0.803	5.0 (1–8)	0.591	1.053	13.0 (3–28)	0.760	0.548
	No	152	4.0 (1–12)			4.0 (1–8)			4.0 (1–8)			13.0 (3–27)		
Education	Yes	107	5.0 (1–12)	0.004 **	11.050	4.0 (1–8)	0.271	2.612	5.0 (1–8)	0.910	0.189	13.0 (6–26)	0.822	0.393
	No	144	4.0 (1–12)			4.0 (1–8)			4.0 (1–8)			13.0 (3–28)		

A *p*-value < 0.05 was considered statistically significant (* *p* < 0.05; ** *p* < 0.01).

## Data Availability

The original contributions presented in the study are included in the article; further inquiries can be directed to the corresponding author.

## Supplementary Materials

The following supporting information can be downloaded at https://www.mdpi.com/article/10.3390/healthcare13131512/s1, Table S1: Comparative analysis of participants’ knowledge scores across three breast cancer subdomains by demographics and lifestyle.

## Abbreviations

The following abbreviations are used in this manuscript:

ASIR	Age-standardized incidence rate
BSE	Breast self-examination
BC	Breast cancer
GBD	Global Burden of Disease
HAPA	Health Action Process Approach
HBM	Health Belief Model
HRT	Hormone replacement therapy
IQR	Interquartile range
M/I	Mortality-to-Incidence Ratio
OECD	Organisation for Economic Co-operation and Development
