# Supplementary material for: Understanding Breast Cancer: Awareness, Risk Factors, and Symptoms Among Female Health Science Students in Hungary
Source: Healthcare (Basel). 2025 Jun 25;13(13):1512. doi: 10.3390/healthcare13131512 (PMC12248933; doi:10.3390/healthcare13131512)
Supplement: Supplementary file 1 [file healthcare-13-01512-s001.zip › healthcare-3615980-supplementary.pdf]

Table S1 Comparative Analysis of Participants' Knowledge Scores Across Three Breast Cancer Subdomains by Demographic and Lifestyle (n = 251)

				Descriptive analysis					Knowledge scores, median (min-max)					
Variables		N	Non-mod risk factors	*p.value*	Mean Rank	Test statistic	Lifestyle risk factors	*p.value*	Mean Rank	Test statistic	Symptoms	*p.value*	Mean Rank	Test statistic
Age group	18-29	211	5.0 (1–12)		126.72		4.0 (1–8)		127.05		4.0 (1–8)		121.87	
	30-41	23	5.0 (1-12)	0.917	120.22	0.173	4.0 (1–8)	0.031*	145.59	6.946	6.0 (1–8)	0.111	147.00	4.393
	42-53	17	4.0 (2–11)		124.94		3.0 (1–5)		86.53		6.0 (3–8)		148.88	
Years of university studies	First year	72	4.0 (1–12)		115.10		3.0 (1–8)		102.42		4.0 (1–8)		115.44	
	Second year	71	4.0 (1–12)	0.101	120.42	4.580	3.0 (1–8)	<0.001***	120.85	15.892	5.0 (1–8)	0.335	130.68	2.186
	Third year	108	5.0 (1–12)		136.94		4.0 (1–8)		145.10		4.5 (1–8)		129.96	
Specialization	Nurse	39	4.0 (1-12)		114.12		3.0 (1-8)		99.94		4.0 (1-7)		95.65	
	Dietetics	25	5.0 (2-12)		129.58		5.0 (1-8)		139.28		5.0 (1-8)		146.62	
	Physiotherapist	84	5.0 (1-12)	0.653	131.92	6.850	4.0 (1-8)	0.033*	135.68	18.203	5.0 (1-8)	0.288	130.19	10.825
	Midwife	28	5.0 (1-11)		128.23		4.0 (2-8)		132.61		4.0 (1-8)		121.39	
	Paramedic	9	5.0 (2-12)		160.06		5.0 (1-8)		159.50		4.0 (2-8)		138.22	

	Public health inspector	4	6.0 (3-10)		146.38		5.5 (5-6)		197.75		5.5 (2-8)		145.13	
	Health visitor	17	5.0 (1-12)		128.00		4.0 (1-8)		118.15		5.0 (2-8)		139.82	
	Recreation	2	6.5 (4-9)		159.00		5.5 (3-8)		165.25		5.5 (3-8)		145.75	
	Laboratory analytics	14	4.0 (2-12)		119.14		3.0 (1-7)		97.43		5.0 (2-8)		129.71	
	Radiography	29	4.0 (1-12)		106.09		3.0 (1-7)		110.59		4.0 (1-8)		123.66	
Perceived financial status	Bad	6	4.0 (3-11)		121.00		2.0 (2-7)		68.17		5.5 (1-8)		136.00	
	Just sufficient	77	5.0 (1-12)	0.011*	144.74	11.091	4.0 (1-8)	0.076	137.53	6.869	5.0 (1-8)	0.953	125.94	0.337
	Good	131	4.0 (1-12)		123.13		4.0 (1-8)		125.06		4.0 (1-8)		127.04	
	Excellent	37	4.0 (1-11)		97.97		4.0 (1-8)		114.70		4.0 (1-8)		120.81	
Level of trust in the healthcare system and physicians	Low	68	4.0 (1-12)		112.60		4.0 (1-8)		128.90		4.0 (1-8)		120.04	
	Moderate	156	5.0 (1-12)	0.028*	135.36	7.163	4.0 (1-8)	0.060	130.07	5.628	4.5 (1-8)	0.592	128.91	1.047
	High	27	3.0 (1-12)		105.67		3.0 (1-7)		95.19		4.0 (1-8)		114.11	
If I had a cancerous disease, I	Not sure	8	4.0 (3-6)	0.004 **	123.94	10.876	5.0 (1-8)	0.127	150.00	4.131	5.0 (2-8)	0.331	148.06	2.214
	Agree	74	4.0 (1-12)		103.09		3.0 (1-8)		112.80		4.0 (1-8)		116.94	

would want to know about it.	Strongly agree	169	5.0 (1-12)		136.13		4.0 (1-8)		130.64		4.0 (1-8)		128.92	
Physical activity	Yes	118	5.0 (1-12)	0.806	127.18	7707.5	4.0 (1-8)	0.020*	137.12	6535.0	4.5 (1-8)	0.936	126.39	7801.5
	No	133	4.0 (1-12)		124.95		3.0 (1-8)		116.14		4.0 (1-8)		125.66	
Alcohol consumption	Yes	211	5.0 (1-12)	0.547	133.86	5141.0	4.0 (1-8)	0.015*	116.40	5040.0	4.0 (1-8)	0.857	121.40	5330.0
	No	40	5.0 (1-12)		123.64		4.0 (1-8)		128.89		4.5 (1-8)		127.38	
Family medical history	Negative	138	4.0 (1-12)	0.025*	116.81	6526.5	4.0 (1-8)	0.726	124.57	7599.5	4.0 (1-8)	0.185	120.57	7047.5
	Positive	113	5.0 (1-12)		137.23		4.0 (1-8)		127.75		5.0 (1-8)		132.63	
Performs self-examination	Yes	178	5.0 (1-12)	0.027*	132.41	5356.0	4.0 (1-8)	0.122	130.47	5700.5	5.0 (1-8)	0.025*	132.49	5341.5
	No	73	4.0 (1-12)		110.37		3.0 (1-8)		115.09		4.0 (1-8)		110.17	
Performed self-examination within the past month	Yes	126	5.0 (1-12)	0.032*	135.68	6655.5	4.0 (1-8)	0.317	130.50	7308.5	5.0 (1-8)	0.053	134.72	6776.5
	No	125	4.0 (1-12)		116.24		4.0 (1-8)		121.47		4.0 (1-8)		117.21	

A p-value <0.05 was considered statistically significant (p < 0.05; ** p < 0.01; *** p < 0.001).*
